# Vegan Diet Is Associated with a Lower Risk of Chronic Kidney Disease in Patients with Hyperuricemia

**DOI:** 10.3390/nu15061444

**Published:** 2023-03-16

**Authors:** Chia-Lin Wu, Wen-Hsin Tsai, Jia-Sin Liu, Hao-Wen Liu, Sin-Yi Huang, Ko-Lin Kuo

**Affiliations:** 1 Department of Post-Baccalaureate Medicine, College of Medicine, National Chung Hsing University, Taichung 402202, Taiwan; 2 School of Medicine, Chung Shan Medical University, Taichung 40201, Taiwan; 3 Division of Nephrology, Department of Internal Medicine, Changhua Christian Hospital, Changhua 50006, Taiwan; 4 Department of Pediatrics, Taipei Tzu Chi Hospital, Buddhist Tzu Chi Medical Foundation, New Taipei City 23142, Taiwan; 5 School of Medicine, Tzu Chi University, Hualien 97004, Taiwan; 6 Division of Nephrology, Taipei Tzu Chi Hospital, Buddhist Tzu Chi Medical Foundation, New Taipei City 23142, Taiwan; 7 Good Clinic, New Taipei City 24146, Taiwan; 8 Division of Family Medicine, Mackay Memorial Hospital, Taipei 104217, Taiwan; 9 School of Post-Baccalaureate Chinese Medicine, Tzu Chi University, Hualien 97004, Taiwan

**Keywords:** acid load, chronic kidney disease, hyperuricemia, plant-based diet, vegan diet, vegetarian diet

## Abstract

Hyperuricemia is a well-known risk factor for chronic kidney disease (CKD). Little is known about whether a vegetarian diet is associated with a lower risk of CKD in patients with hyperuricemia. From 5 September 2005, to 31 December 2016, we retrospectively included clinically stable patients with hyperuricemia who received health check-ups at Taipei Tzu Chi Hospital. All participants completed a dietary habits questionnaire to determine whether they were omnivorous, lacto-ovo vegetarian, or vegan. CKD was defined as an estimated glomerular filtration rate <60 mL/min/1.73 m^2^ or the presence of proteinuria. A total of 3618 patients with hyperuricemia were recruited for this cross-sectional study, consisting of 225 vegans, 509 lacto-ovo vegetarians, and 2884 omnivores. After adjusting for age and sex, vegans had a significantly lower odds ratio (OR) of CKD than omnivores (OR, 0.62; *p =* 0.006). The OR of CKD remained significantly lower in vegans after adjusting for additional confounders (OR, 0.69; *p =* 0.04). Additionally, age (per year OR, 1.06; *p* < 0.001), diabetes mellitus (OR, 2.12; *p* < 0.001), hypertension (OR, 1.73; *p* < 0.001), obesity (OR, 1.24; *p* = 0.02), smoking (OR, 2.05; *p* < 0.001), and very high uric acid levels (OR, 2.08; *p* < 0.001) were independent risk factors for CKD in patients with hyperuricemia. Moreover, structural equation modeling revealed that a vegan diet was associated with a lower OR of CKD (OR, 0.69; *p* < 0.05). A vegan diet is associated with a 31% lower risk of CKD in patients with hyperuricemia. A vegan diet may be beneficial in reducing the occurrence of CKD in patients with hyperuricemia.

## 1. Introduction

Chronic kidney disease (CKD) is a large global health burden [1]. CKD accounts for approximately 10–15% of the population in most nations around the world. In 2008, a large epidemiological surveillance in Taiwan, which screened up to 462,293 participants, showed that approximately 12% of the Taiwanese population had CKD [2]. In general, CKD is an irreversible disorder. Progressive loss of nephrons exaggerates glomerular hyperfiltration and the activation of the renin–angiotensin–aldosterone system, leading to renal fibrosis, and end-stage renal disease (ESRD) inevitably develops. Patients with CKD and ESRD will have substantially higher rates of mortality and cardiovascular or cerebrovascular morbidities. These patients who survived morbidities would still have prolonged years lived with disability. It is more important to prevent the development of CKD than to treat the disease. Common risk factors for CKD include aging, male sex, hypertension, diabetes mellitus, obesity, and hyperuricemia [3]. Among these conditions, hypertension, diabetes mellitus, and obesity are modifiable risk factors, but aging and gender are not correctable ones. Since the prevalence of CKD increases worldwide, it is emerging as important to search for novel modifiable risk factors of CKD and their corresponding treatments.

Hyperuricemia is a disorder of abnormally high uric acid in the blood. In general, hyperuricemia can be diagnosed in individuals with a serum uric acid level equal to or greater than 7 mg/dL [4]. Uric acid is the end product of purines and is almost freely filtrated by the glomerulus in humans [5]. Then, uric acid is reabsorbed and secreted via the proximal renal tubule, and ~10% filtrated uric acid appears in the final urine [6]. Hyperuricemia can result from reduced urinary excretion, increased production of uric acid, or both. Hyperuricemia and gout are more common in patients with impaired kidney function.

Studies have shown that hyperuricemia can cause or accelerate the progression of CKD [7,8]. A previous study prospectively followed 21,475 healthy volunteers for a median of seven years and reported that a slightly elevated serum uric acid level (7–8.9 mg/dL) was associated with an odds ratio (OR) of 1.74 for incident CKD, and a markedly elevated uric acid level (greater than 9 mg/dL) was linked to a triple risk [7]. A recent basic study reported that hyperuricemia per se did not cause CKD, but hyperuricemia with crystalluria led to CKD and drove progressive CKD [9]. This means that the crystallization of uric acid in the urine is crucial for the development and progression of CKD in patients with hyperuricemia. Because uric acid is a weak acid with a *pK_a_* of 5.75, it appears mainly as monosodium urate in the urine at physiologic pH [5]. However, the solubility of uric acid decreases with decreasing urine pH, and low urine pH can exaggerate uric acid crystallization. Although emerging evidence reveals a causal relationship between hyperuricemia and CKD, urate-lowering therapy remains controversial in patients with asymptomatic hyperuricemia.

The debate continues about whether a vegetarian diet is healthier than an omnivorous diet. Vegetarian or plant-based diets may have multiple beneficial effects on our bodies, though they could also contribute to the deficiency of nutrients, such as vitamin B12, vitamin D, iron, calcium, zinc, and ω-3 fatty acids [10]. Studies have reported that a vegetarian diet is associated with a lower risk of gout and hyperuricemia [11,12]. In addition, dietary habits can affect urine pH. Omnivores have lower urine pH and higher net acid excretion than vegetarians [13]. The situation may promote uric acid appearance with crystallization in the urine. Otherwise, the crystallization of uric acid in the renal tubule or interstitium would cause kidney damage and result in CKD or the progression of kidney disease.

However, it is unknown whether vegetarian dietary habits are beneficial for kidney health in patients with hyperuricemia. Thus, this study aimed to assess the association of vegetarian dietary habits with CKD in patients with gout or hyperuricemia.

## 2. Materials and Methods

### 2.1. Participants

**Figure 1:**
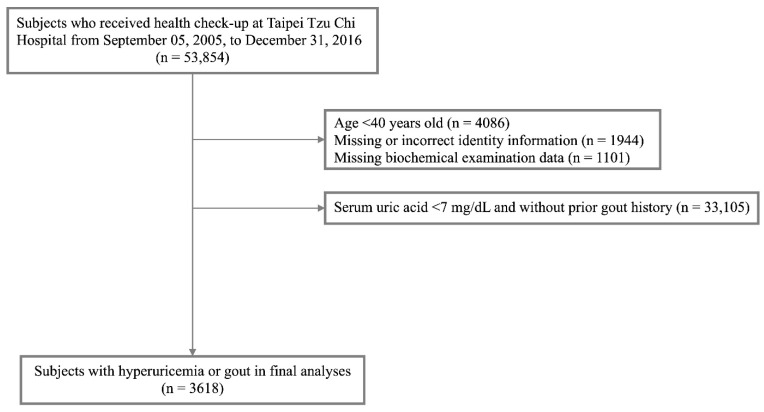
CONSORT diagram of the study. From 5 September 2005, to 31 December 2016, 53,854 participants had a health check-up at Taipei Tzu Chi Hospital. We excluded participants younger than 40 years (*n* = 4086), participants without hyperuricemia or a diagnosis of gout (*n* = 33,105), participants with incorrect or incomplete identity information (*n* = 1944), and participants with missing biochemistry data (*n* = 1101). Finally, 3618 participants with hyperuricemia or gout entered the final analyses. CONSORT, consolidated standards of reporting trials.

We retrospectively included 53,854 participants who had a health check-up at Taipei Tzu Chi Hospital from 5 September 2005, to 31 December 2016. We excluded 4086 participants younger than 40 years (they may have distinct pathogenesis for their kidney disease, e.g., polycystic kidney disease, lupus nephritis, type 1 diabetes mellitus, or congenital anomalies), 33,105 participants without hyperuricemia or a diagnosis of gout, 1944 participants with incorrect or incomplete identity information (such as foreigners, registration errors, or missing values), and 1101 participants with missing biochemistry data. Thus, 3618 participants with hyperuricemia or gout were included in the final analyses (Figure 1). The study was approved by the Institutional Review Board of Taipei Tzu Chi Hospital (approval number: 06-XD12-033). Written informed consent was waived. This research is strictly adherent to the Declaration of Helsinki and the guidelines for academic ethics by Taiwan’s Ministry of Science and Technology.

### 2.2. Outcome Measures

Hyperuricemia was defined by a serum uric acid concentration of greater than 7.0 mg/dL. The primary endpoint was CKD, which was defined as an estimated glomerular filtration rate (eGFR) of less than 60 mL/min/1.73 m^2^ or the presence of proteinuria [14].

### 2.3. Clinical and Biochemical Measurements

All subjects completed structured dietary questionnaires [15,16,17] and underwent a comprehensive health examination. The dietary questionnaire on dietary practices in terms of type and duration as well as a quantitative food frequency questionnaire has been validated in previous studies [16,17]. The food frequency questionnaire consists of 64 food items, which are categorized into 10 food groups, with additional sections on the consumption of beverages and dietary supplements. A trained research nurse interviewed every participant to assess their age, sex, vital signs, body mass index (BMI), medical history, lifestyle (smoking, alcohol consumption, and physical activity), and dietary habits from the dietary questionnaire at the entry of the study. Obesity in this Taiwanese adult population was defined as a BMI of >27 kg/m^2^ (based on the criteria by Taiwan’s Ministry of Health and Welfare) [18]. The subjects were grouped according to their self-reported dietary habits as vegans (only consumed plant-based foods), lacto-ovo vegetarians (ate eggs and/or dairy products but no other animal products), and omnivores (ate both plant- and animal-based foods) [15,16]. Height, weight, and blood pressure were measured by using an electronic meter (SECA GM-1000, Seoul, Republic of Korea) and an automatic blood pressure machine (Welch Allyn 53000, New York, NY, USA), respectively.

Blood sampling was performed after a 12 h fasting period. Serum uric acid, creatinine (Jaffe’s reaction), glycated hemoglobin (HbA1c), albumin, low-density lipoprotein cholesterol, and high-density lipoprotein cholesterol were measured by using a biochemistry analyzer (Dimension^®^ RXL Max^®^ integrated chemistry system, Siemens, Erlangen, Germany). The eGFR was calculated by using the equation from the Chronic Kidney Disease Epidemiology Collaboration (CKD-EPI) [19]. We evaluated urine protein semi-quantitatively with a dipstick test (AX-4030, Arkray Inc., Tokyo, Japan), as we described previously [15]. Proteinuria was classified into 6 ordinal categories: absent (<10 mg/dL), trace (10 to 30 mg/dL), 1+ (30 mg/dL), 2+ (100 mg/dL), 3+ (300 mg/dL), or 4+ (1000 mg/dL). Patients with trace levels, 1+ levels, or more were determined to have proteinuria.

### 2.4. Statistical Analyses

Data are presented as the number (percentage) or mean (standard deviation, SD) as appropriate. The means and proportions among the groups were compared by one-way analysis of variance (ANOVA) and Chi-square tests, respectively. In addition, Fisher’s exact test was used to compare the distribution among the groups instead of the Chi-square test if there was an observed value of <5 with regard to categorical variables. We used logistical regression to determine the crude OR of dietary habits (omnivorous, lacto-ovo vegetarian, and vegan diets) with the primary endpoint. Moreover, the crude OR was adjusted for traditional risk factors of CKD, such as age, sex, diabetes mellitus, hypertension, obesity, smoking, and uric acid [3]. The crude association was further adjusted for age and sex (Model 2) and for other well-known risk factors for CKD (diabetes mellitus, hypertension, BMI, current smoking, and uric acid; these confounders were also statistically significant in Model 3) in multivariate logistical regression models. Bayesian logistic regression was conducted for multiplicity correction. Furthermore, multilevel structural equation modeling (SEM) with a multivariate Bernoulli distribution and logit link function by logistic regression was used to estimate the direct and indirect effects of dietary habits and other variables on the risk of CKD in patients with hyperuricemia. This SEM was limited to specific patterns and presented the factor–variable notation in path diagrams, which included the main risk factors and their endogenous effects. Two levels of relationships were shown, including (1) the direct effects of all potential risk factors on CKD and (2) the indirect effects of dietary habits on those potential CKD risk factors. This model was identified, and these constraints are supplied. In accordance with the above conditions, the estimation of SEM by method of maximum likelihood was performed using the GSEM package of STATA. Sensitivity analysis was performed using the multiple imputation and the expectation–maximization (EM) algorithm. A two-tailed *p*-value of less than 0.05 was considered statistically significant. All statistical analyses were performed by using SAS software (version 9.4, SAS Institute Inc., Cary, NC, USA) and STATA (version 15.1, Stata Corp, College Station, TX, USA).

## 3. Results

### 3.1. Patient Characteristics

A total of 3618 patients with hyperuricemia were grouped into 2884 omnivores, 509 lacto-ovo vegetarians, and 225 vegans (Table 1). Compared with the omnivore group, the vegan group was older; had a lower proportion of smoking, alcohol drinking, obesity, gout, and very high uric acid levels (>9 mg/dL); and had lower diastolic blood pressure and uric acid, low-density lipoprotein, and creatinine levels.

### 3.2. Associations between Dietary Habits and CKD in Patients with Hyperuricemia

We used logistic regression models to reveal the associations between the self-reported dietary behaviors and CKD in this cross-sectional research. Univariate logistic regression showed that age (OR, 1.06; 95% confidence interval (CI), 1.05–1.07, *p* < 0.001), diabetes mellitus (OR, 3.04; 95% CI, 2.46–3.77, *p* < 0.001), hypertension (OR, 2.69; 95% CI, 2.31–3.14, *p* < 0.001), obesity (OR, 1.20; 95% CI, 1.03–1.41, *p* = 0.02), and uric acid level >9 mg/dL (OR, 1.92; 95% CI, 1.55–2.38, *p* < 0.001) were associated with higher ORs for CKD in patients with hyperuricemia (Table 2). In contrast, male sex was associated with a lower OR for CKD (OR, 0.71; 95% CI, 0.61–0.83, *p* < 0.001). After adjusting for age and sex (Model 2, Table 2), a vegan diet was associated with a lower risk of CKD in patients with hyperuricemia (OR, 0.62; 95% CI, 0.45–0.97, *p* = 0.006) compared with the omnivore diet. Moreover, after adjusting for all potential risk factors in the fully adjusted model (Model 3), the association between a vegan diet and the primary outcome remained statistically significant (OR, 0.69; 95% CI, 0.48–0.99, *p* = 0.04). We also used Bayesian logistic regression for multiplicity correction. After correction, the association was consistent with the result in Model 3 (vegan vs. omnivore: OR, 0.71; equal-tailed 95% confidence interval, 0.50 to 0.94).

### 3.3. Interactive Effects of Potential Risk Factors for CKD in Patients with Hyperuricemia

**Figure 2:**
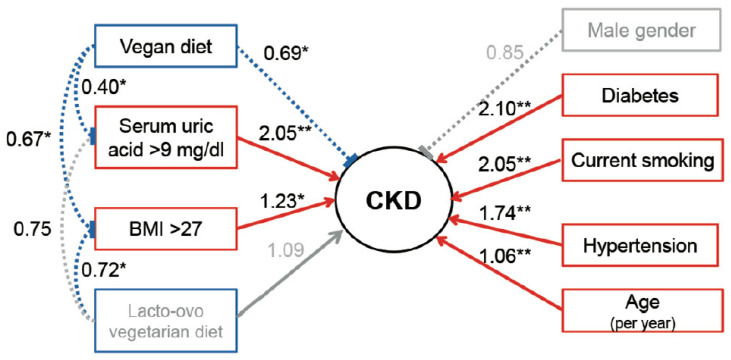
The odds ratios of potential risk factors for CKD in the multilevel structural equation modeling. Odds ratios are shown near the lines indicating the association between the variables and outcome. Blue dotted lines indicate significantly reduced odds ratios. Red solid lines indicate significantly higher odds ratios. Grey solid or dotted lines indicate non-significant associations. BMI, body mass index. CKD, chronic kidney disease. * *p* value < 0.05. ** *p* value < 0.001.

We conducted SEM analysis to further investigate the effects between a vegetarian diet and other potential risk factors on the ORs for CKD in the path diagram (Figure 2). The specific patterns in the path diagram presented the estimated and compared effects of results in our identified SEM. A vegan diet was linked not only to a lower OR (0.69, *p* < 0.05) but also to lower ORs for serum uric acid levels >9 mg/dL (0.40, *p* < 0.05) and BMIs greater than 27 (0.67, *p* < 0.05), which were both associated with higher ORs for CKD (2.05, *p* < 0.001 and 1.23, *p* < 0.05, respectively). A lacto-ovo vegetarian diet was not significantly associated with the OR for CKD or serum uric acid level of greater than 9 mg/dL but was significantly linked to a lower OR for obesity, defined as BMI >27 (0.72, *p* < 0.05).

In addition, diabetes mellitus (OR, 2.10, *p* < 0.001), smoking (OR, 2.05, *p* < 0.001), hypertension (OR, 1.74, *p* < 0.001), and age (OR per year, 1.06, *p* < 0.001) had higher ORs for CKD but did not significantly interact with a vegetarian diet. Compared with Model 3, the SEM analysis has shown that the effect was significant in the goodness-of-fit through the test of the Log likelihood ratio (Log likelihood from −1826.17 to −5351.02, *p* < 0.001).

### 3.4. Sensitivity Analysis

We excluded 3045 subjects before the final analysis (Figure 1). Therefore, we conducted a sensitivity analysis by using multiple imputations and the EM algorithm. Table 3 shows the result of the sensitivity analysis, which was similar to the result of the primary analysis in Table 2.

## 4. Discussion

For the first time, we found that a vegan, but not a lacto-ovo vegetarian, diet was independently associated with a lower OR for CKD in patients with hyperuricemia. Other risk factors for CKD in patients with hyperuricemia include age, diabetes mellitus, hypertension, obesity, smoking, and very high serum uric acid levels (>9 mg/dL). Moreover, a vegan diet was linked to lower risks for very high uric acid levels and obesity, which are two important risk factors for CKD.

Plant-based diets, defined as vegan and lacto-ovo-vegetarian diets, are growing in popularity worldwide [20]. Plant-based diets do not necessarily eliminate animal products but focus on eating mostly plants, such as fruits, vegetables, nuts, seeds, and whole grains. Plant-based diets also highlight eating whole foods without much processing that are as close to their natural state as possible. Plant-based diets are beneficial for metabolic health over animal-based diets [21]. CKD patients consuming plant-based proteins had a lower rate of disease progression or mortality [22,23]. Consistent with previous studies, our research showed that vegan or lacto-ovo vegetarian diets were associated with lower ORs (0.4 and 0.72, respectively) for obesity, which was further associated with a higher OR for CKD. This suggests that the renoprotective effect of a vegetarian diet in patients with hyperuricemia may be due partly to the improvement of metabolic overload. In addition, hyperuricemia could lead to uric acid stone formation and CKD [9,24]. Very high (more than 9 mg/dL) serum uric acid tripled the OR for incident CKD [7]. Our research also found that a serum uric acid level >9 mg/dL doubled the OR for CKD in patients with hyperuricemia. Furthermore, a lower dietary acid load may also favorably affect insulin resistance, insulin sensitivity, glycemic control, and other factors associated with CKD [25]. Vegan or plant-based diets have been shown to have a lower dietary acid load burden and increase 24 h urine pH [26,27,28]. Alkali treatment with vegetables and fruits has been shown to increase plasma total carbon dioxide and preserve the eGFR in patients with Stage 3 CKD [29]. Thus, patients with hyperuricemia consuming a vegan diet may have less crystallization of uric acid in the urine and a lower risk for subsequent CKD.

The association of hyperuricemia with vegetarians and omnivores remains controversial. Gajski et al. reported no significant difference in uric acid levels between vegetarians and omnivores [30]. Another study found that vegans had higher serum uric acid concentrations than meat eaters [31]. Similar to our study, Szeto et al. found that vegetarians had lower uric acid concentrations than omnivores [12]. In addition, few studies have reported that lacto-ovo vegetarians have lower risks of hyperuricemia or gout [11,15]. Unlike our previous study [15], a lacto-ovo vegetarian diet was not significantly linked to lower ORs for very high serum uric acid levels and CKD in patients with hyperuricemia. Although high-level dairy product consumption is associated with a lower risk of gout in men [32], our result shows that vegetarians eating egg and dairy products may not have significantly lower risks of hyperuricemia and CKD. We need further prospective, large-scale, longitudinal studies to clarify the actual impacts of the lacto-ovo dietary habit on serum uric acid levels and CKD.

Our study concurs with the previous findings that traditional risk factors of CKD, such as older age, diabetes mellitus, hypertension, obesity, smoking, and a very high level of serum uric acid, were significant risk factors for CKD in patients with hyperuricemia [3]. It is possible that these risk factors contribute to CKD in patients with hyperuricemia and in other populations through similar mechanisms. In contrast, our study shows that the male sex was associated with a 17% lower risk for CKD in patients with hyperuricemia. Women have a higher prevalence of CKD according to previous population-based studies [33,34]. However, it is possible that the use of the CKD-EPI equation (which has not been widely validated in the Taiwanese population) to determine the eGFR resulted in the overdiagnosis of CKD in women because the measurement bias could lead to significant underestimation of the eGFR and misclassification in women [35]. In addition, women have longer life expectancy with a natural decrease in GFR during aging [33]. In the current study, we used the presence of proteinuria in conjunction with a reduced creatinine-based eGFR value to determine the diagnosis of CKD. Further studies are needed to elucidate the sex effect on CKD in patients with gout or hyperuricemia. Using a new creatinine- and cystatin C-based eGFR equation may also be needed to reduce the bias from the estimation of GFR by equations [36].

Hypertension, diabetes mellitus, and obesity are well-known modifiable risk factors for CKD. For decades, blood pressure control with angiotensin-converting enzyme inhibitors or angiotensin II receptor blockers, strict blood glucose control, and weight control have been shown to prevent the development of CKD or disease progression. To date, whether hyperuricemia is a modifiable risk factor continues to be unclear. Although growing evidence has shown a significant relationship between hyperuricemia and CKD and the pathogenesis of uric acid nephropathy, treatment of asymptomatic hyperuricemia with urate-lowering medications is still controversial around the world. Recently, a meta-analysis also revealed that urate-lowering treatment did not slow the progression of CKD in patients with asymptomatic hyperuricemia [37]. Since the role of pharmacological treatments in patients with hyperuricemia remains unclear, our results will shed some light on non-pharmacological interventions for the prevention of incident CKD or its progression. Whether a vegan diet prevents the development of CKD in patients with hyperuricemia is worthy of further study by a randomized controlled trial or a large-scale cohort study in the future.

Plant-based diets have many additional benefits to our health. First, many vegetables are rich in antioxidants, such as vitamins A (e.g., carrots), C (e.g., broccoli), and E (e.g., spinach), carotenoids (e.g., tomatoes and carrots), phenolic acids (e.g., potatoes), and flavonoids (e.g., onions) [38]. These antioxidants might enhance immune activity and reduce inflammatory responses to pathogens and chronic diseases. Thus, the lower risk for CKD associated with a vegan diet in patients with hyperuricemia may be at least partly attributed to consuming antioxidant-rich vegetables. Second, most vegetables are also rich in dietary fiber. An increase in the consumption of dietary fiber is proposed to prevent the development or to slow the progression of CKD for decades. Chiavaroli et al. reported that dietary fiber supplements could decrease blood urea and creatinine concentrations in a meta-analysis of controlled trials [39]. Their results may also support the possible underlying mechanism of the renoprotective role of a vegan diet in patients with hyperuricemia in addition to ameliorating the crystallization of uric acid in the kidney. Third, recent studies have shown that a plant-based diet could improve gut microbiota to mitigate oxidative stress and inflammation and reduce uremic toxins in patients with CKD [40]. Gut microbiota might also be a therapeutic target to prevent CKD in our study population. Further studies are needed to clarify the underlying mechanisms of the renoprotective effect of a vegan diet in patients with hyperuricemia.

There are limitations of this study. First, the cross-sectional study design cannot determine the causal relationship between the vegan dietary habit and the subsequent development and progression of CKD in our study population. Second, the dietary habits were self-reported. Their actual eating habits were not confirmed by professional dieticians. However, the food frequency questionaries in the current study included detailed food items and categories and could easily be conducted by a research nurse. This method has been validated in previous studies [16,17]. Third, based on the retrospective study design, there may be unmeasurable confounding factors not included in the adjustment models. Forth, retrospective analysis of secondary data excluded 3045 patients with missing information who might be eligible for analysis. This may increase the uncertainty of the association between the primary study variable of interest and outcome. Fifth, the creatinine-based CKD-EPI equation may lead to an underestimation of the eGFR and may not be the optimal equation for determining the presence of CKD in Taiwanese women. Sixth, some changes to the equipment for anthropometric and blood pressure measurements and laboratory tests had been made during the long study period, although all equipment has been validated by the Taiwan Food and Drug Administration. Finally, the current study recruited participants from a single medical center and mainly from the Han Taiwanese population, and our results might not be generalizable to other nations or ethnicities.

In conclusion, a self-reported vegan dietary habit but not a lacto-ovo vegetarian diet was significantly associated with lower risks of obesity and very high serum uric acid levels. Moreover, patients with hyperuricemia consuming a vegan diet had a 31% lower risk for CKD. Prospective cohort studies are warranted to confirm our results.

## Figures and Tables

**Table 1:** The characteristics of participants with hyperuricemia in the study.

Characteristics	Vegans (*n* = 225)	Lacto-Ovo Vegetarians (*n* = 509)	Omnivores (*n* = 2884)	*p*-Value
**Demographics**				
Age group, years old, *n* (%)				<0.001 ^a^
40–49, *n* (%)	12 (5.3)	52 (10.2)	474 (16.4)	
50–69, *n* (%)	40 (17.8)	159 (31.2)	838 (29.1)	
60–69, *n* (%)	99 (44.0)	193 (37.9)	920 (31.9)	
>70, *n* (%)	74 (32.9)	105 (20.6)	652 (22.6)	
Age, years old, mean (SD)	66.3 (10.6)	62 (9.4)	61.3 (11.4)	<0.001 ^b^
Gender				<0.001 ^a^
Male, *n* (%)	116 (51.6)	282 (55.4)	2100 (72.8)	
Female, *n* (%)	109 (48.4)	227 (44.6)	784 (27.2)	
**Comorbid conditions**				
Current smoking, *n* (%)	0 (<0.1)	4 (0.8)	173 (6.0)	<0.001 ^a^
Alcohol drinking, *n* (%)	17 (7.6)	44 (8.6)	1066 (37.0)	<0.001 ^a^
BMI, Kg/m^2^, mean (SD)	25.2 (3.8)	25.3 (3.8)	25.9 (3.8)	<0.001 ^b^
>27, *n* (%)	56 (24.9)	133 (26.1)	951 (33.0)	0.001 ^a^
Hypertension, *n* (%)	86 (38.2)	179 (35.2)	1075 (37.3)	0.73 ^a^
SBP, mmHg, mean (SD)	129 (18)	125 (15)	127 (16)	0.59 ^b^
DBP, mmHg, mean (SD)	77 (12)	76 (11)	79 (12)	0.001 ^b^
Diabetes mellitus, *n* (%)	23 (10.2)	51 (10.0)	322 (11.2)	0.77 ^a^
Gout, *n* (%)	101 (44.9)	195 (38.3)	1383 (48.0)	<0.001 ^a^
CKD, *n* (%)	51 (23.1)	133 (26.3)	744 (25.9)	0.55 ^a^
Stage 3, *n* (%)	39 (17.6)	112 (22.2)	602 (20.9)	0.35 ^a^
Stages 4–5, *n* (%)	3 (1.4)	9 (1.8)	49 (1.7)	0.91 ^a^
**Biochemistry**				
Uric acid, mg/dL, mean (SD)	7.3 (1.3)	7.5 (1.2)	7.8 (1.4)	<0.001 ^b^
>9 mg/dL, *n* (%)	12 (5.3)	48 (9.4)	353 (12.2)	0.002 ^a^
HbA1c, %, mean (SD)	5.8 (0.7)	5.8 (1.0)	5.8 (1.0)	0.64 ^b^
HDL-C, mg/dL, mean (SD)	42.1 (10.3)	44.3 (12.0)	44.1 (12.7)	0.50 ^b^
LDL-C, mg/dL, mean (SD)	118.6 (32.4)	122.2 (30.3)	129.9 (34.9)	<0.001 ^b^
Serum albumin, mg/dL, mean (SD)	4.3 (0.4)	4.2 (0.4)	4.3 (0.4)	0.006 ^b^
Serum creatinine, mg/dL, mean (SD)	0.9 (0.3)	1.0 (0.4)	1.0 (0.6)	0.001 ^b^
CKD-EPI eGFR, mL/min/1.73 m^2^, mean (SD)	76 (17)	77 (17)	77 (17)	0.51 ^b^
Proteinuria, *n* (%)	30 (13.6)	77 (15.2)	423 (14.7)	0.84 ^a^

Abbreviations: BMI, body mass index; CKD, chronic kidney disease; CKD-EPI, Chronic Kidney Disease Epidemiology Collaboration; DBP, diastolic blood pressure; HbA1c, hemoglobin A1c; HDL-C, high-density lipoprotein cholesterol; LDL-C, low-density lipoprotein cholesterol; SBP, systolic blood pressure; SD, standard deviation. ^a^ Chi-square test. ^b^ One-way ANOVA test.

**Table 2:** Risks for chronic kidney disease in participants with hyperuricemia (*n* = 3618).

	Model 1 ^a^		Model 2 ^b^		Model 3 ^c^	
	Odds Ratio (95% CI)	*p*-Value	Odds Ratio (95% CI)	*p*-Value	Odds Ratio (95% CI)	*p*-Value
Dietary habits						
Vegan vs. omnivores	0.86 (0.62–1.19)	0.36	0.62 (0.45–0.97)	0.006	0.69 (0.48–0.99)	0.04
Lacto-ovo vegetarian vs. omnivores	1.02 (0.83–1.27)	0.83	0.99 (0.78–1.25)	0.99	1.15 (0.91–1.45)	0.23
Age, per year	1.06 (1.05–1.07)	<0.001	1.06 (1.05–1.07)	<0.001	1.06 (1.05–1.06)	<0.001
Male gender	0.71 (0.61–0.83)	<0.001	0.89 (0.56–0.86)	0.19	0.83 (0.70–0.99)	0.04
Diabetes mellitus	3.04 (2.46–3.77)	<0.001			2.12 (1.68–2.67)	<0.001
Hypertension	2.69 (2.31–3.14)	<0.001			1.73 (1.45–2.05)	<0.001
BMI > 27 kg/m^2^	1.20 (1.03–1.41)	0.02			1.24 (1.04–1.47)	0.02
Current smoking	1.28 (0.92–1.78)	0.14			2.05 (1.44–2.94)	<0.001
Serum uric acid > 9 mg/dL	1.92 (1.55–2.38)	<0.001			2.08 (1.64–2.63)	<0.001
SBP, per 10 mmHg	1.22 (1.16–1.28)	<0.001				
HbA1c, %	1.17 (1.09–1.26)	<0.001				
Hyperlipidemia	1.00 (0.99–1.02)	0.63				
LDL-C, per 10 mg/dL	0.98 (0.95–1.01)	0.20				
Alcohol drinking	1.28 (0.92–1.78)	<0.001				

Abbreviations: BMI, body mass index; CI, confidence interval; HbA1c, hemoglobin A1c; LDL-C, low-density lipoprotein cholesterol; SBP, systolic blood pressure. ^a^ Crude model. ^b^ Adjusted for age and gender. ^c^ Full adjusted model: adjusted for the variables in Model 2 and diabetes mellitus, hypertension, BMI, current smoking, uric acid, lacto-ovo vegetarian diet, and vegan diet.

**Table 3:** Risks for chronic kidney disease in participants with hyperuricemia after using the multiple imputation method and EM algorithm.

	Model 1 ^a^		Model 2 ^b^		Model 3 ^c^	
	Odds Ratio (95% CI)	*p*-Value	Odds Ratio (95% CI)	*p*-Value	Odds Ratio (95% CI)	*p*-Value
Dietary habits						
Vegan vs. omnivores	0.85 (0.61–1.17)	0.31	0.59 (0.42–0.82)	0.002	0.65 (0.46–0.93)	0.02
Lacto-ovo vegetarian vs. omnivores	1.08 (0.88–1.33)	0.45	1.03 (0.83–1.27)	0.8	1.21 (0.97–1.51)	0.09
Age, per year	0.88 (0.83–0.93)	<0.001	1.06 (1.05–1.06)	<0.001	0.86 (0.81–0.91)	<0.001
Male gender	0.58 (0.48–0.69)	<0.001	0.75 (0.63–0.91)	0.003	0.76 (0.63–0.93)	0.006
Diabetes mellitus	3.03 (2.46–3.73)	<0.001			2.12 (1.69–2.66)	<0.001
Hypertension	2.52 (2.18–2.91)	<0.001			1.63 (1.38–1.91)	<0.001
BMI > 27 kg/m^2^	1.13 (0.97–1.31)	0.11			1.13 (0.95–1.33)	0.16
Current smoking	1.35 (1.02–1.79)	0.033			2.10 (1.56–2.84)	<0.001
Serum uric acid >9 mg/dL	1.99 (1.61–2.46)	<0.001			2.00 (1.59–2.52)	<0.001
SBP, per 10 mmHg	1.21 (1.16–1.26)	<0.001				
HbA1c, %	1.26 (1.13–1.41)	<0.001				
Hyperlipidemia	1.00 (0.99–1.02)	0.63				
LDL-C, per 10 mg/dL	0.97 (0.94–1.00)	0.07				
Alcohol drinking	0.73 (0.63–0.85)	<0.001				

Abbreviations: BMI, body mass index; CI, confidence interval; HbA1c, hemoglobin A1c; LDL-C, low-density lipoprotein cholesterol; SBP, systolic blood pressure. ^a^ Crude model. ^b^ Adjusted for age and gender. ^c^ Full adjusted model: adjusted for the variables in Model 2 and diabetes mellitus, hypertension, BMI, current smoking, uric acid, lacto-ovo vegetarian diet, and vegan diet.

## Data Availability

The datasets used or analyzed during the current study are available from the corresponding author upon reasonable request.
